# Letter to the Editor on "Functional outcomes after retrosigmoid approach to the cerebellopontine angle: Observations from a single-center experience of over 13 years"

**DOI:** 10.1016/j.bas.2024.102924

**Published:** 2024-08-13

**Authors:** Ayush Anand, Muhammed Shabil, Nitin Kumar Bansal, Sanjit Sah, Olabisi Oluwagbemiga Ogunleye

**Affiliations:** BP Koirala Institute of Health Sciences, Dharan, Nepal; Center for Global Health Research, Saveetha Medical College and Hospital, Saveetha Institute of Medical and Technical Sciences, Saveetha University, Chennai, India; Evidence for Policy and Learning, Global Center for Evidence Synthesis, Chandigarh, India; Department of General Medicine, Graphic Era (Deemed to Be University), Clement Town, Dehradun, 248002, India; Department of Paediatrics, Dr. D. Y. Patil Medical College, Hospital and Research Centre, Dr. D. Y. Patil Vidyapeeth, Pune, 411018, Maharashtra, India; Department of Public Health Dentistry, Dr. D.Y. Patil Dental College and Hospital, Dr. D.Y. Patil Vidyapeeth, Pune, 411018, Maharashtra, India; Neurosurgery, Department of Surgery, Abubakar Tafawa Balewa University Bauchi, Nigeria

Dear Editor,

The retrosigmoid approach hailed as the workhorse for accessing the cerebellopontine angle (CPA), remains a cornerstone in the neurosurgical management of various pathologies such as vestibular schwannomas, meningiomas, and trigeminal neuralgia (Basma et al., 2022). The study titled “Functional outcomes after retrosigmoid approach to the cerebellopontine angle: Observations from a single-center experience of over 13 years," authored by Aftahy et al., presents a comprehensive analysis of 517 patients treated using the retrosigmoid approach (RSA) (Aftahy et al., 2024). This study provides invaluable insights into the functional outcomes and complications associated with this widely utilized neurosurgical technique.

The findings underscore several critical points that can refine surgical practice and improve patient outcomes. First, one of the notable findings is the association between larger craniotomy sizes and increased postoperative complications, particularly cerebrospinal fluid (CSF) leaks (Aftahy et al., 2024). This is a significant revelation, as it emphasizes the necessity for precision in the surgical approach. Surgeons must carefully balance the need for adequate exposure with the risks associated with larger craniotomies. Also, the focus should be on improving preoperative planning, potentially utilizing advanced imaging techniques and intraoperative neuronavigation to minimize craniotomy size without compromising surgical efficacy. Second, the study also highlights the relatively high complication rates associated with the RSA, including CSF leaks, postoperative hemorrhage, and hydrocephalus (Aftahy et al., 2024). These complications, particularly CSF leaks, have a domino effect, often leading to further complications such as meningitis and the need for revision surgeries (Oh et al., 2017; Sathaporntheera et al., 2020). Addressing these complications involves meticulous surgical technique and postoperative care. For instance, ensuring watertight dural closure using modern sealants and grafts can significantly reduce the incidence of CSF leaks (Oh et al., 2017). Third, the functional outcomes reported in the study, with a substantial number of patients experiencing permanent symptom improvement, reaffirm the efficacy of the RSA (Aftahy et al., 2024). However, new permanent neurological deficits in two-fifths of patients cause concern (Aftahy et al., 2024). This evidence suggests that while the approach is practical, there is considerable room for improvement in minimizing collateral damage. Intraoperative neurophysiological monitoring can be crucial in this regard, helping preserve cranial nerve function during tumor resection (Guzzi et al., 2024).

Despite its extensive data and detailed analysis, the study by Aftahy et al. has inherent limitations typical of retrospective analyses. First, one primary limitation is the study's non-interventional, retrospective design, which cannot establish causality. Prospective, randomized controlled trials (RCTs) are needed to confirm the findings and provide more definitive evidence. Second, the heterogeneity of the patient population, encompassing various pathologies and tumor entities, also complicates the interpretation of results. Future studies should aim to stratify patients based on specific pathologies and conduct subgroup analyses to derive more nuanced insights. For instance, separating outcomes for vestibular schwannomas from meningiomas could help tailor surgical approaches and postoperative care specific to each pathology. Third, another significant limitation is the relatively short median follow-up period of ten months. Long-term follow-up is crucial in assessing the durability of surgical outcomes and the late onset of complications. Developing comprehensive databases capturing a wide array of clinical, radiological, and surgical parameters would facilitate more detailed analyses and better generalizability of findings. Fourth, relying on the Karnofsky Performance Status Scale (KPSS) and other subjective measures for evaluating functional outcomes may introduce bias. Incorporating objective measures, such as quantitative audiometric data for hearing outcomes and detailed neurocognitive assessments, would enhance the robustness of the findings. In addition, innovation in surgical technology, such as the integration of augmented reality and robotic-assisted surgery, holds promise for improving the precision and safety of the RSA. These technologies can assist in preoperative planning, intraoperative navigation, and real-time monitoring, potentially reducing the risk of complications and improving functional outcomes.

In conclusion, while the study by Aftahy et al. provides valuable insights into the RSA's functional outcomes and complications, there is a pressing need for further research to refine surgical techniques, enhance patient selection, and optimize postoperative care. By addressing the limitations of current studies and leveraging technological advancements, the neurosurgical community can continue to improve the safety and efficacy of the retrosigmoid approach, ultimately enhancing the quality of life for patients with CPA pathologies.

## Ethical approval

Not applicable.

## Consent to participate

Not applicable.

## Consent for publication

Not applicable.

## Funding

This research received no specific grant from any funding agency in the public, commercial, or not-for-profit sectors.

## Availability of data and materials

Not applicable.

## Author contribution

Ayush Anand = Writing-original draft and Writing-review and editing.

Muhammed Shabil = Writing-original draft and Writing-review and editing.

Nitin Kumar Bansal = Writing-original draft and Writing-review and editing.

Sanjit Sah = Writing-original draft and Writing-review and editing.

## Declaration of competing interest

The Author(s) declare(s) that there is no conflict of interest.
